# Comparison between adherence assessments and blood glucose monitoring measures to predict glycemic control in adults with type 1 diabetes: a cross-sectional study

**DOI:** 10.1186/s13098-016-0162-4

**Published:** 2016-07-29

**Authors:** Gabriela Heiden Telo, Martina Schaan de Souza, Thais Stürmer Andrade, Beatriz D’Agord Schaan

**Affiliations:** 1 Internal Medicine Department, Universidade Federal do Rio Grande do Sul, Rua Ramiro Barcellos, 2350, Porto Alegre, CEP 90035-903 Brazil; 2 Endocrine Division, Hospital de Clínicas de Porto Alegre, Porto Alegre, Brazil

**Keywords:** Diabetes mellitus, type 1, Medication adherence, Blood glucose monitoring

## Abstract

**Background:**

Adherence to treatment has been defined as the degree to which a patient’s behavior corresponds to medical or health advice; however, the most appropriate method to evaluate adherence to diabetes care has yet to be identified. We conducted analyses to compare adherence assessments and blood glucose monitoring measures with regard to their ability to predict glycemic control in adults with type 1 diabetes.

**Methods:**

We analyzed four instruments to evaluate adherence: Self-Care Inventory-Revised, a self-administered survey; Diabetes Self-Monitoring Profile (DSMP), administered by trained researchers; a categorical (yes/no/sometimes) adherence self-evaluation; and a continuous (0–100) adherence self-evaluation. Blood glucose monitoring frequency was evaluated by self-report, diary, and meter download.

**Results:**

Participants (n = 82) were aged 39.0 ± 13.1 years with a mean diabetes duration of 21.2 ± 11.1 years; 27 % monitored blood glucose >4 times/day. The DSMP score was the strongest predictor of glycemic control (*r* = −0.32, P = 0.004) among adherence assessments, while blood glucose monitoring frequency assessed by meter download was the strongest predictor among blood glucose monitoring measures (*r* = −40, P < 0.001). All the self-report assessments had a significant but weak correlation with glycemic control (*r* ≤ 0.28, P ≤ 0.02). The final adjusted model identified the assessment of blood glucose monitoring frequency by meter download as the most robust predictor of HbA1c (estimate effect size = −0.58, P = 0.003).

**Conclusions:**

In efforts to evaluate adherence, blood glucose monitoring frequency assessed by meter download has the strongest relationship with glycemic control in adults with type 1 diabetes.

## Background

Adherence to treatment has been defined as the degree to which a patient’s behavior corresponds to medical or health advice [1]. Despite all evidence that achieving good glycemic control helps prevent microvascular and macrovascular complications of diabetes, many patients do not achieve such control, mostly because treatment adherence is poor [2, 3]. Sustained glycemic control has been shown to be difficult in adults of all ages, as the management of diabetes places substantial demands on patients [4]. Challenges to adherence and active patient engagement in diabetes care include, but are not limited to, physical and emotional barriers, complex treatment regimens, and financial burdens [5]. There is a tendency in the literature to treat adherence and glycemic control as interchangeable constructs [6], while, in fact, patient adherence and metabolic control need to be assessed both independently and concomitantly [6, 7]. Patients in good glycemic control cannot be presumed to be adherent.

In the literature and in clinical practice, there are various methods of assessing adherence to diabetes care, such as structured interviews, self-report, diaries, and electronic monitoring [8]. Many of these methods have been shown to correlate well with glycemic control; however, some traditional methods, such as insulin bottles count at the pharmacy, may not be suitable enough as an adherence measure in type 1 diabetes considering that many patients should change their daily insulin dose based on sliding scales and carb counting, which may interfere in adherence interpretation [9]. On the other hand, surveys have been validated and widely used as measures to evaluate adherence in this population [10, 11]. Their domains usually capture behavioral characteristics related to diabetes management, such as insulin administration, meal plans, frequency/intensity of exercises, frequency of blood glucose monitoring, and hypoglycemia [6]. The appropriate execution of all these tasks was shown to promote optimal glycemic control [2, 3], which solidified the association between adherence and metabolic results in diabetes treatment. In contrast, several studies have demonstrated the importance of focusing on specific adherence behaviors, such as blood glucose monitoring [12, 13]. These studies demonstrated strong association between a higher frequency of blood glucose monitoring and lower hemoglobin A1c (HbA1c) levels. However, the most appropriate method to evaluate adherence to diabetes care in adults with type 1 diabetes has yet to be identified.

In this study, we sought to investigate different methods of assessing adherence and glycemic control. To accurately predict HbA1c in adults with type 1 diabetes, we designed a cross-sectional study to evaluate and compare adherence assessments by structured surveys and self-report, as well as blood glucose monitoring measures by self-report, diary, and electronic devices. Such knowledge may enhance opportunities to better understand this important barrier and assist in development of strategies to improve adherence to diabetes care and glycemic control in patients with type 1 diabetes.

## Research design and methods

### Participants

We conducted exploratory multivariable analyses to compare adherence assessments and blood glucose monitoring measures with regard to their ability to predict HbA1c in adults with type 1 diabetes on injection therapy. All participants included in these analyses met the following inclusion criteria: ≥18 years of age and type 1 diabetes duration ≥1 year. Exclusion criteria included a developmental disability or a psychiatric disorder that would interfere with reliable completion of the structured instruments. We selected patients from the outpatient electronic medical record database of a single tertiary public hospital in Southern Brazil. The Institutional Review Board approved the study protocols, and all participants signed informed consent forms prior to beginning any study procedure.

### Measures

#### Adherence assessments

We analyzed four different instruments to evaluate adherence to diabetes management. First, participants were asked to respond to a three-level (no/sometimes/yes) categorical self-report question (“In the past month, did you take care of your diabetes as your doctor recommended?”). Patients were also asked to characterize themselves according to their adherence to diabetes care on a continuous self-evaluation scale, ranging from 0 to 100. Additionally, all participants completed the two following previously validated adherence surveys:

- Self-Care Inventory-Revised version (SCI-R) [11, 14]: the 14-item SCI-R [14] is a self-administered survey, which measures adherence to different diabetes management tasks on a 5-point Likert scale. Responses range from 1 = never to 5 = always, and scores range from 14 to 70. Higher scores indicate greater adherence to type 1 diabetes treatment.
- Diabetes Self-Monitoring Profile (DSMP) [10, 14]: the DSMP [14] is a 24-item survey administered by trained researchers, which measures adherence to 5 different domains: exercises, hypoglycemia, diet, blood glucose tests, and insulin dose. Scores range from 0 to 96. Higher scores indicate greater adherence to type 1 diabetes treatment.

#### Blood glucose monitoring measures

We evaluated blood glucose monitoring frequency by three different ways: self-report, diary, and meter download. Participants were asked to take their blood glucose meters and blood glucose diary to the study visit. An average frequency of blood glucose monitoring per day was calculated for the last 14 days, not including the visit day.

After collecting responses to the adherence assessments and blood glucose monitoring data, trained researchers interviewed participants to obtain additional clinical and demographic data. Socioeconomic status was characterized based on the *Associação Brasileira de Empresas de Pesquisa* criteria [15], which includes data regarding costumer goods and parental education. Glycemic control was assessed by HbA1c, which was performed in a clinical laboratory using a Diabetes Control and Complications Trial standardized assay (high-performance liquid chromatography, ref. range 4.0–6.0 %).

### Data analysis

Analyses were performed using SAS software (version 9.3; Institute, Inc., Cary, NC, USA). Descriptive data are presented as mean ± standard deviation (SD) or percentage. Statistical analyses included Pearson correlations to determine associations between adherence assessments and blood glucose monitoring measures. Exploratory multivariable analyses were conducted using stepwise regression to identify, among all adherence assessments and blood glucose monitoring measures, the best predictor of HbA1c. Three different steps were performed in the stepwise analyses: first, all three blood glucose monitoring assessments were included in the HbA1c model; second, all four adherence assessments were included in the HbA1c model; and, finally, after selecting the best HbA1c predictors among the adherence assessments and blood glucose monitoring measures based on the two first steps, a final step evaluated the two selected measures in order to elucidate which one could better predict HbA1c. Generalized linear model was then performed, including mean HbA1c as the dependent variable and the best adherence measure based on the final exploratory step as the independent variable. Multivariable analyses were performed to evaluate the impact of demographics and clinical characteristics on the adherence-glycemic control relationship. Also, as insulin regimen may interfere with adherence to diabetes treatment, multivariable analyses included this variable to evaluate its impact on the results. An alpha level of <0.05 was used to determine statistical significance. The Cohen’s index was used to determine correlation coefficients and effect size [16]. A sample size of 82 was calculated as sufficient to detect a moderate effect size between HbA1c and adherence measures considering an alpha of 0.05 and power of 80 %.

## Results

### Participant characteristics

In total, 103 eligible patients were approached to participate in this study (from March 2014 to September 2014), of those 82 (80 %) agreed to participate. All participants provided written informed consent. Those who declined participation had similar age, diabetes duration, and HbA1c as those who agreed to participate (P > 0.05). All participants provided data regarding blood glucose meter and diary and responded to the study visit interview and surveys. Participants had a mean age of 39.0 ± 13.1 years and mean diabetes duration of 21.2 ± 11.1 years; 39 % were overweight/obese, 27 % had a frequency of blood glucose monitoring >4 times/day according to the study visit interview (see Table 1), and 82 % were under flexible insulin regimens, such as sliding scale and carb counting. All participants in this study were using daily multiple injections for diabetes treatment and only 11 % met the ADA HbA1c target of <7 % [17].

**Table 1:** Demographic and clinical characteristics of study participants

	N = 82
Age (years)	39.0 ± 13.1
Sex (% male)	63
Race/ethnicity (% Caucasian)	98
Socioeconomic class (%)	
High	13
Medium	70
Low	15
Very low	2
Diabetes duration (years)	21.2 ± 11.1
Blood glucose monitoring (% ≥4 times/day)	27
HbA1c (%)	8.9 ± 2.2
HbA1c (mmol/mol)	74 ± 24
Daily insulin dose (units/kg)	0.74 ± 0.30
Weight status (% overweight/obese)	39

Data are mean ± SD or n (%)

### Measures results

All adherence assessments appeared to be interrelated (P < 0.01), as did the blood glucose monitoring measures (P < 0.001). The correlations between DSMP score and blood glucose monitoring frequency assessed by self-report (*r* = 0.69, P < 0.001) and meter download (*r* = 0.52, P < 0.001) were identified as the two strongest correlations between the adherence and blood glucose monitoring measures (see Table 2).

**Table 2:** Correlation between adherence and blood glucose monitoring assessments

Assessments	Blood glucose monitoring frequency (self-report) (*r*)	Blood glucose monitoring frequency (diary) (*r*)	Blood glucose monitoring frequency (meter collection/downloading) (*r*)
Self-administered survey (SCI-R)	0.50	0.46	0.40
P value	<0.0001	<0.0001	0.0002
Structured interview/survey (DSMP)	0.69	0.32	0.52
P value	<0.0001	0.004	<0.0001
Self-report (categorical)	0.31	0.28	0.41
P value	0.005	0.01	0.0001
Self-report (continuous)	0.34	0.25	0.33
P value	0.002	0.02	0.003

*SCI-R* Self Care Inventory-Revised, *DSMP* Diabetes Self-Monitoring Profile

Exploratory analyses using stepwise multivariable regression were conducted to identify the best predictor of HbA1c based on all the adherence and blood glucose monitoring measures. In the first step, among all the blood glucose monitoring measures, frequency of blood glucose monitoring assessed by meter download was the strongest predictor of HbA1c (*r* = −40, P < 0.001) (see Table 3a). In the second step, among all the adherence assessments, DSMP score was the strongest predictor of glycemic control (*r* = −0.32, P = 0.004) (see Table 3b). During the two first steps, all adherence and blood glucose assessments were significantly correlated with HbA1c (P < 0.05), except for frequency of blood glucose monitoring by diary (*r* = −0.20, P = 0.07). All self-report assessments, which included the blood glucose monitoring frequency self-report, categorical adherence self-evaluation question, and continuous adherence self-evaluation question, had a weak, although significant, correlation with glycemic control. In the final step, which included the best adherence and blood glucose monitoring assessments based on the two first steps, frequency of blood glucose monitoring by meter download emerged as the most robust predictor of glycemic control (*r* = −33, P < 0.001). The DSMP score, in this final model, was no longer significant (*r* = −15, P = 0.22).

**Table 3:** Stepwise regression for HbA1c and adherence assessments

Assessments	R^2^	P value	*β*
(a) Blood glucose monitoring assessments			
Blood glucose monitoring frequency (self-report)	0.08	0.01	−0.28
Blood glucose monitoring frequency (diary)	0.04	0.07	−0.20
Blood glucose monitoring frequency (meter collection/downloading)	0.16	0.0002	−0.40
*Blood glucose monitoring frequency (meter collection/downloading)*	*0.16*	*0.0002*	*−0.40*
(b) Adherence-specific assessments			
Self-report (categorical)	0.06	0.02	−0.27
Self-report (continuous)	0.07	0.01	−0.27
Self-administered survey (SCI-R)	0.05	0.04	−0.23
Structured interview/survey (DSMP)	0.10	0.004	−0.32
*Structured interview/survey (DSMP)*	*0.10*	*0.004*	*−0.32*
(c) Final model	0.18		
Structured interview/survey (DSMP)		0.22	−0.15
Blood glucose meter collection/downloading		0.007	−0.33
*Blood glucose meter collection/downloading*	*0.16*	*0.0002*	*−0.40*

Italic values indicate variables selected by the stepwise regression

*SCI-R* Self Care Inventory-Revised, *DSMP* Diabetes Self-Monitoring Profile, *β* standardized estimate

Generalized linear model was initially performed to evaluate the interaction between HbA1c and blood glucose monitoring frequency assessed by meter download. This analysis showed that as frequency of blood glucose monitoring increased 1 time/day, HbA1c decreased 0.63 % (P < 0.001). The final adjusted model (P < 0.001), which controlled for age, diabetes duration, insulin dose, insulin regimen, and socioeconomic status, slightly decreased the adherence-glycemic control association (estimate effect = −0.59, P = 0.003). Demographics and clinical characteristics were not significant in this model (P > 0.05). As DSMP may also be an alternative while assessing adherence in clinical settings, we also performed univariate analysis to evaluate the interaction between HbA1c and DSMP scores. In this analysis, as DSMP score increased by 10 points, HbA1c decreased by 0.74 % (P = 0.004).

## Discussion

The conceptual and methodological issues related to adherence assessments are important for research but also have widespread clinical application. How healthcare providers conceptualize adherence impacts diabetes management recommendations [6]. In order to better understand the different instruments available to assess adherence in adults with type 1 diabetes, we sought to evaluate and compare adherence assessments and blood glucose monitoring measures to predict glycemic control. In this study, among the four instruments to assess adherence (DSMP, SCI-R, and categorical and continuous self-evaluations), DSMP score was the strongest predictor of glycemic control, while blood glucose monitoring frequency assessed by meter download was the most robust predictor of HbA1c among the blood glucose monitoring measures and all the studied assessments.

Our findings highlight the observation that there is a strong association between frequency of blood glucose monitoring and glycemic control in a population of patients with type 1 diabetes. Indeed, the T1D Exchange Clinic Network [13] clearly demonstrated that for all ages, increased frequency of blood glucose monitoring is associated with lower HbA1c. Previous studies also identified that capillary glucose information was valuable for making appropriate decisions with regard to insulin doses [18]. This is true even after adjusting for demographics and socioeconomic confounders [13]. In this study, demographics and diabetes clinical characteristics did not appear to have an impact on the adherence-glycemic control relationship, although it is true that some methods used to evaluate demographics such as socioeconomic status may not be enough accurate and may underestimate its impact on the results [15]. Also, we know that frequent blood glucose monitoring by itself does not directly impact HbA1c; the capillary blood glucose information must be used effectively in diabetes management in order for the frequency of monitoring to impact glycemic control [5, 19]. Thus, frequency of blood glucose monitoring seems to be a behavior strongly representative of adherence and very well associated with glycemic control.

Besides frequency of blood glucose monitoring, many other domains seem to be related to adherence and have been assessed by different surveys [10, 11]. However, despite the well-known importance of adherence in achieving good glycemic control and preventing diabetes complications [3, 20], there are a few questionnaires with established psychometric properties to assess it [10, 11, 21, 22]. In this study, we evaluated the two instruments validated in Brazilian Portuguese to assess adherence in patients with type 1 diabetes [14]. Both surveys, as well as the self-evaluation questions, demonstrated significant correlation with glycemic control and seem to be appropriate to evaluate adherence in adults with type 1 diabetes. The DSMP, which includes domains regarding diet, exercises, insulin, blood glucose monitoring frequency, and hypoglycemia, showed to have the most powerful questions to predict glycemic control when compared to self-evaluations. However, it is essential to state that, although surveys are an easy-to-use instrument to assess adherence, blood glucose measures assessed by meter download appear to have the strongest relationship with HbA1c in adults with type 1 diabetes. Surveys may have utility for periodic use with patients in clinical and research settings [9]; however, although surveys can predict glycemic control and health outcomes, they cannot be interpreted as substitutes of HbA1c and must be analyzed with consideration of the clinical setting. Interestingly, in this study, all the self-report assessments, including the SCI-R, had a significant but weak correlation with glycemic control. Consistent with previous studies [6, 13], adherence assessments by self-evaluation may over-report engagement in diabetes tasks and frequency of blood glucose self-monitoring.

It is important that we do not overestimate our findings. This study involved a cross-sectional design, and our results represent associations, not direct causal relationships, between glycemic control and adherence measures. Moreover, as occurs frequently in behavioral studies, we were reliant on self-reports that were not confirmed objectively. Further studies may want to confirm our findings in longitudinal research designs, while controlling for the timeframe of assessing adherence behaviors. Moreover, future studies need to be designed to better evaluate the impact of demographic characteristics on adherence in order to best predict glycemic control.

## Conclusions

To our knowledge, this is the first study designed to compare different methods to assess adherence and blood glucose monitoring frequency in adults with type 1 diabetes. Our findings highlight blood glucose monitoring frequency by meter download as the best method to assess adherence and glycemic control. The knowledge from this study stresses the importance of downloading blood glucose monitoring devices and provides opportunities to better understand and assess adherence to diabetes management. Appropriately identifying modifiable characteristics associated with glycemic control may aid providers as they consider a diabetes prescription or management plan. Further studies are necessary to determine the role of each adherence assessment in diabetes care longitudinally.
